# A Multimodal Approach to Improve Performance Evaluation of Call Center Agent

**DOI:** 10.3390/s21082720

**Published:** 2021-04-12

**Authors:** Abdelrahman Ahmed, Khaled Shaalan, Sergio Toral, Yasser Hifny

**Affiliations:** 1 Department of Electronic Engineering, University of Seville, 41092 Seville, Spain; abdahm@alum.us.es; 2 Faculty of Engineering & IT, British University in Dubai, Dubai 345015, United Arab Emirates; Khaled.shaalan@buid.ac.ae; 3 Faculty of Computers and Artificial Intelligence, University of Helwan, Helwan 11795, Egypt; yhifny@fci.helwan.edu.eg

**Keywords:** performance modeling, multimodal classification, BiLSTM, CNNs, attention layer

## Abstract

The paper proposes three modeling techniques to improve the performance evaluation of the call center agent. The first technique is speech processing supported by an attention layer for the agent’s recorded calls. The speech comprises 65 features for the ultimate determination of the context of the call using the Open-Smile toolkit. The second technique uses the Max Weights Similarity (MWS) approach instead of the Softmax function in the attention layer to improve the classification accuracy. MWS function replaces the Softmax function for fine-tuning the output of the attention layer for processing text. It is formed by determining the similarity in the distance of input weights of the attention layer to the weights of the max vectors. The third technique combines the agent’s recorded call speech with the corresponding transcribed text for binary classification. The speech modeling and text modeling are based on combinations of the Convolutional Neural Networks (CNNs) and Bi-directional Long-Short Term Memory (BiLSTMs). In this paper, the classification results for each model (text versus speech) are proposed and compared with the multimodal approach’s results. The multimodal classification provided an improvement of (0.22%) compared with acoustic model and (1.7%) compared with text model.

## 1. Introduction

Evaluating the performance of call-center agents involves several issues. The first is that the evaluation is performed manually by listening to recorded calls and evaluating the content, which most likely will be a subjective evaluation [1,2]. Proficiency in oral communications is an essential skill in call centers, and it is very important for fulfilling the customers’ needs. However, the customer service representative tone, oral proficiency, communications, and listening skills are most likely subjective factors that cause a bias in the evaluation process [3,4,5]. The second issue is that the number of calls is huge over a while, i.e., one year, which makes the manual evaluation very challenging. Hence, the evaluation is performed randomly over selected calls out of thousands of records. This can lead to missing the more realistic performance that occurred during the majority of the calls. The third obstacle is the diversity of the evaluators so that they may rank the same agent’s performance differently. The lack of a unified system of evaluation can have a significant adverse impact on the business of call centers when the baseline is overlooked. Avoiding subjectivity and automating performance evaluation is essential in reducing the time and effort associated with the manual evaluation process. It leads to the establishment of a call center’s performance baseline with a unified system of evaluation.

Objective methods in performance evaluation have been developed to overcome the subjective factors and assessment bias [6,7]. A discussion is presented for the two studies that considered the binary classification for either the text or speech of the recorded calls [8]. The productivity classification differs significantly when using speech processing instead of the text approach. There is a massive number of speech features that can be extracted from the recorded calls in comparison to the features of the text [9]. Furthermore, the text-based approach requires a minimum word error rate (WER) for the transcription system for better classification accuracy. However, more sophisticated data extraction and computational resources for speech modeling are required than for the text approach.

The research framework of this study is a multimodal classification based on different approaches that combine text and speech processing for improving accuracy. The proposed multimodal approach is the main paper contribution to empower the classification accuracy when combining the best classification performance obtained for speech and text. The models comprise different neural network structures to classify the speech utterances side by side with the corresponding call transcribed text into productive and nonproductive classes (binary classification). The study attempts to determine the best accuracy by combining the three techniques using the attention layer, Max Weights Similarity (MWS), and the multimodal system to improve the performance evaluation. Also, we investigated the performance of MWS compared with the Softmax function, which may reflect on the accuracy of the classification and encourage future studies.

The rest of the paper is structured as follows: Section 2 discusses the works related to performance evaluation in call centers. The study framework is illustrated in Section 3. The experiment and results are stated in Section 4. Finally, Section 5 concludes the paper and suggests the future research avenues.

## 2. Related Work

Many studies of machine learning are concerned with the processing of speech to detect the eminent factors of customer behavior and causes of complaints [10]. Other studies were concerned with analytics to detect the service quality based on the content of the recorded calls from customers. That framework uses Hadoop Map Reduce for text distances using Cosine distance and n-gram supported by slang words [11]. Perera et al. [12] studied the automatic performance evaluation of call center agents. They determined various factors to improve the performance evaluations, such as the speech utterances, the tone level, and the emotional characteristics, then classified using the support vector machine (SVM). Sudarsan et al. examined several systems to evaluate the performance based on prohibited words, emotional recognition, and others [13]. Their framework was based on platforms like Google, Wit, and Sphinx for transcription. Ahmed et al. [7] transcribed the text based on lexicon free Recurrent Neural Networks (RNN) supported by Connectionist Temporal Classification (CTC) objective function [14]. They annotated the corpus into productive/nonproductive and modeled the text using one generative approach (Naive Bayes), and two discriminative approaches (logistical regression and linear support vector machine (LSVM)) [6,7]. The generative and discriminative approaches were modeled on a bag of words as text features.

The emotion recognition and speech enhancement studies have an important exposure for behavior determination, and classification improvement [15,16,17,18]. They intended to enhance the speech quality to measure human behavior’s emotional aspects and gestures based on sophisticated deep neural network training. Performance evaluation extends these studies by determining the performance from a human conversation. This study focuses on the call scenarios considering performance as the core part of the call center processes. The call center gives two advantages to the study: mixing the natural conversation between two parties and the high regularity in the conversation path over a high volume of calls. The call follows a standard and predefined script like welcoming message, agent name, and services [19,20].

Both CNNs and LSTMs dominate the deep learning approaches, and they have provided outstanding improvements in various studies [16,21]. Named entity recognition (NER) is a cascaded CNNs-LSTMs approach to extract critical medical information from electronic medical records [22]. The convolutional layers extract the prominent features in a fast and restricted manner. The long-short-term memory layers (LSTMs) are intended to handle the long sequential streams of inputs. The attention layer uses the Softmax function followed by the weighted average vector (context vector) and forwarded to the classifier to improve the accuracy [23,24].

Ahmed et al. explored the productivity measurement using speech signal processing [25]. The study was performed using MFCC 13 features extracted and forwarded to different combinations of CNNs and LSTMs structures. The attention layer was applied to enhance the binary classification up to 84.27%, which means an improvement of around 1.57% over text classification approaches. Besides, the attention weights highlighted the para-linguistic features where the productivity measurement takes place. Yet, there were issues associated with using MFCC features for the binary classification problem. MFCC only provides information about the vocal track; it ignores prosodic information. Therefore, providing MFCCs to CNNs is very restrictive and limits the ability of the CNN to use discriminative features for better classification. Accordingly, in this study, extended speech features were used to overcome the previous studies’ limitations and improve the classification accuracy by combining the text and the speech models. The next sections demonstrate how several modeling approaches are combined to improve the model’s accuracy and compare the previous text and speech classification approaches.

## 3. The Proposed Framework

**Figure 1:**
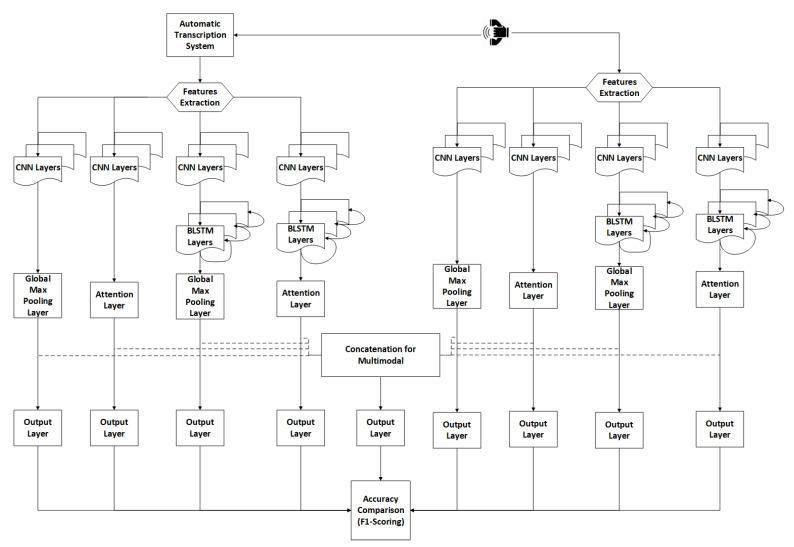
The proposed framework illustrates two schemes for speech and text. The dotted lines indicate the multimodal approach for merging one path for each scheme and forward it to the output layer.

There are several alternatives for modeling speech and text. In this study, two main schemes are proposed for modeling text and speech, as shown in Figure 1. The first branch is for modeling speech using CNNs, cascaded CNNs-LSTMs, and an attention layer. Each branch of the speech modeling presents one or more of the deep learning combinations, i.e., CNNs, CNNs-attention, CNNs-LSTMs, and CNNs-LSTMs-Attention layers. The features extraction has been extended to 65 features using the Open-smile toolkit [26]. Open-smile is a comprehensive toolkit for the extraction of audio and music features, and it supports low-level audio descriptors, such as Mel-frequency cepstral coefficients (MFCC), fundamental frequency, formant frequencies, perceptual linear predictive cepstral coefficients, CHROMA, and CENS features, loudness, line spectral frequencies, and linear predictive coefficients. The frames are forwarded to the four branches of speech modeling to get the best accuracy compared with other speech models. The text is transcribed by using an automatic speech recognition system with Word Error Rate (WER) 12.03% [8]. The transcribed text has been revised and edited manually to avoid WER that affects the text classification accuracy. The extraction of features uses a word embedding layer of approximately 4k vocabulary size (4930 words). The text branch follows the same speech neural network structure to attain the best accuracy for the four branches of the text. The models with the best accuracies for speech and text are then merged (concatenated) at the last neural network layer for sigmoid binary classification.

### 3.1. CNNs and BiLSTMs

CNNs are widely used in signal processing, and speech recognition tasks [27]. The CNNs help scan the extracted features’ frames to obtain the best classification accuracy through the filters. This study considers two main branches as shown in Figure 1: one for the text features and another for the speech features. Each main branch is in turn divided into four subbranches that follows a similar scheme: Two of them make use of 1D-CNNs layers with tanh activation functions followed by either a max-pooling layer or an attention layer, and the other two make use of a 1D-CNN-BiLSTMs also followed by either a max-pooling layer or an attention layer. Finally, a logit sigmoid output layer performs the binary classification into a productive or nonproductive call. The combinations of different models were used in this study to identify the best classification performance.

### 3.2. Attention Layer

The sequence of vectors (frames) produced from CNN or LSTM and forwarded to the attention layer to convert them into a context vector [23,28,29]. The attention weight are forwarded to Softmax function at time *t* to generate the probability of the frame out of one to the remaining frames in the same speech segment. Then the context vector is generated by the weighted average of the frames probabilities. For each vector, $x_{t}$ in a sequence of inputs, i.e., $x_{1},x_{2},\ldots ,x_{T}$, and the attention weights, $\alpha_{t}$, are given by:

(1) $$\alpha_{t}=\frac{\exp(f\left(x_{t}\right))}{\sum_{j=1}^{T}\exp\left(f\left(x_{j}\right)\right)}$$

where $f(x_{t})$ is presented by the parameter w as follows:

(2) $$f\left(x_{t}\right)=\tanh\left(w^{T}x_{t}\right)$$

The weighted average of the Softmax generated weights and the input vector are summed to get the context vector *C*.

(3) $$C=\sum_{t=1}^{T}\alpha_{t}x_{t}$$

The Dense layer *D* uses tanh activation function given by:

(4) $$D=tanh(W^{T}C+b)$$

Being *W* are the hidden layers weights, and *b* is the bias. The Logit function is the output layer for two classes (productive/nonproductive).

(5) y=Logit(D)

### 3.3. Max Weights Similarity (MWS)

The attention layer uses the Softmax function to determine the probability of the hidden layer weights among each other [23]. The Softmax function converts a vector of real values into probability values that sum up to one [30]. Sometimes, the Softmax function is referred to as multi-class logistic regression or the Softargmax function. For the speech processing branch, the wide variety in features (65 features × 25 ms frame, 10 ms frameshift) means that the Softmax can perform efficiently. However, in the text classification part, using a few embedded words limits its efficacy, so the classification accuracy is lower than in the speech processing branch. It can be explained because, in the text classification, the generated context vectors have values quite close to each other, so the attention layer does not have enough variability to reach a value that has a significant accuracy. The study proposes the Max Weights Similarity (MWS) function instead of the Softmax function to overcome the previous limitation. MWS aims to collapse the training weights around a reference value, which is the maximum value of the vector. The MWS function determines the similarity between the maximum value in the vector and the remaining values in the same vector. For each vector $x_{t}$ in a sequence of inputs $x_{1},x_{2},\ldots ,x_{T}$, and $f(x_{t})$ in Equation (2), the attention weights $\alpha_{t}$ and maximum value $\beta_{t}$ of the vector are given by:

(6) $$\beta_{t}=\max\left(\exp\left(f\left(x_{1}\right)\right),\exp\left(f\left(x_{2}\right)\right),\ldots ,\exp\left(f\left(x_{T}\right)\right)\right)$$

The cosine similarity equation for vectors *a* and *b* is as follows:

(7) $$Cosine\_Similarity=\frac{a*b}{∥a∥*∥b∥}$$

The wights $\alpha_{t}$ of the context vector *C* in Equation (3) are given by:

(8) $$\alpha_{t}=\frac{\exp\left(f\left(x_{t}\right)\right)*\beta }{∥\exp\left(f\left(x_{t}\right)\right)∥*∥\beta ∥}$$

Where $∥\exp\left(f\left(x_{t}\right)\right)∥$ is the normalized value of the vector of weights.

Then, the maximum value of the vector is chosen to give significant attention to the values of the vector compared with others. MWS will be applied either on the speech branch to compare its efficiency with the Softmax function.

### 3.4. Multimodal Approach

Many studies have been developed for deep-learning multimodal approaches [31,32,33]. In this study, merging the speech and text models are concatenated at the final layer for classification. The joint representation multimodal approach in [34] is applied to keep the speech and text features separated in the modeling process. Also, it gives a clear picture of the effect of using the multimodal compared with the same models trained alone. Figure 1 shows the dotted lines indicating the merging layer that combines the two speech and text models from the main branch. More specifically, different combinations of speech and text models are merged until achieving the best accuracy. Then, the five cross-validations and F1-scoring are applied for validation. In Equation (4), a merged dense layer concatenates the dense activation output from text and speech branches in Equation (9).

(9) $$D_{Merged\_Dense}=Concatenate\left(D_{Speech},D_{Text}\right)$$

Then forward the merged dense to the classifier in Equation (5). The Dense size is the total number of units for both speech and text layers.

## 4. The Experiment

**Figure 2:**
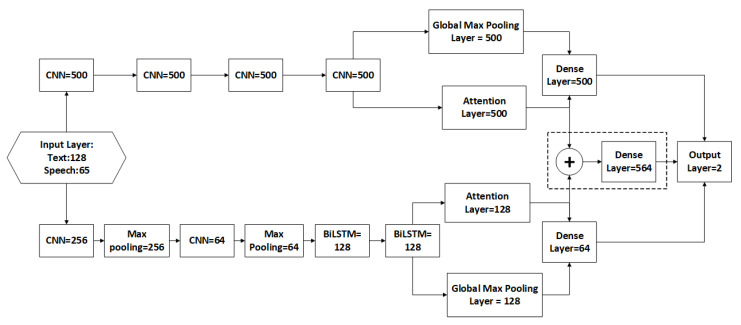
The Study Neural Networks Structure Units.

The experiment is performed over three stages, i.e., speech processing, text processing, and multi-model classification (indicated by dotted box). Five-folds cross-validation with F1-scoring was used to validate the proposed experiments. The training was performed using Nvidia GPUs. The classification models were performed using Tensor-Flow backend and Keras APIs. Figure 2 summarizes the neural network parameters used in the proposed scheme of Figure 1.

The neural network hyper-parameters are set following the configuration defined in the Interactive Emotional Dyadic Motion Capture Database (IEMOCAP) [35], which is followed by various previous studies in emotional recognition and performance measurement [16,25].

### 4.1. The Data

Ethical approval has been granted for collecting the experiment corpus for research purposes from a real estate call center located in Egypt. A call recording system built-in VoIP call center was used between years 2014 and 2015 to collect real calls over landline phones with a sampling rate of 8 kHz. The selected random calls consist of seven hours over 30 calls (14 min per call on average), which is considered adequate compared to similar studies [16,25]. The corpus comprises six different agents between 25–35 years old; two females and four males. The calls were diarized, which is an algorithm to split the voice stream into smaller chunks. Speaker diarization is the process of splitting the speakers’ utterances into separate segments [36] previously in [25] so that the talking time is 40% for females and 60% for males. The naming convention of the recorded calls is built from the metadata as Date, Time, Agent ID, Speaker ID (by the diariser), the call direction, Inbound, Outbound (The wave file name appears like DATE-TIME_AGENT-ID_SPK-ID_CALL-DIRECTION(INBOUND-OUTBOUND).wav). Three independent raters conducted a manual annotation process. The manual annotation may impact or bias the results because of the subjective performance evaluation of the raters. Hence, Krippendorff’s Alpha is used to validate the agreement of the raters that should be more than 80%, which is achieved (Alpha > 0.79 in this study) [20].

### 4.2. Speech Processing

The Open-Smile toolkit for feature extraction [26] can be used to collect 65 features. It is based on INTERSPEECH 2016 Computational Paralinguistics Challenge (2016 COMPARE) [37]. It includes energy-related, spectral-related, and Low-Level Descriptors (LLDs); including logarithmic harmonic-to-noise ratio (HNR), spectral harmonicity, and psychoacoustic spectral sharpness. The features are stated in Table 1. The resulting accuracy from training and validating the models are detailed in Table 2. The accuracy is compared with the previous study [25] in which 13 MFCC features were used.

There was a significant improvement in speech classification using LLD than the previous study (MFCC), with the highest improvement being 8.4%. The attention layer supported with MWS gives a slight improvement of 0.2% for CNN compared with Softmax but about 0.18% less accuracy for CNN-LSTM.

### 4.3. Text Processing

The word embedding layer has been applied to indexed words for the transcribed Arabic text. The generated dictionary is around 4k words with a max stream length of 128 words. The same deep learning structure in Figure 1 was applied with the attention layer using Softmax and MWS. The results were compared with the results of previous experiments of text classification [7] using Logit and SVM based on a bag of words. The results are reported in Table 3.

The deep learning text classification using the embedding of words shows a significant improvement of 8.7% over the generative and discriminative approaches using the bag of words. The MWS has higher accuracy than Softmax only for the CNNs approach (0.42%). (The same happened in the case of the speech approach). The CNNs-BiLSTM is less accurate than the CNNs-Attention model, which coincides with the results of a previous study [25]. It occurred because the BiLSTM is more efficient for long data streams, which is not the case in the short conversations in a call center. Accordingly, the attention layer does not provide a significant classification improvement in CNNs-BiLSTMs compared with CNNs.

### 4.4. Multimodal Approach (Speech + Text)

This step is required to increase the classification accuracy by combining (merging) the models at the final layer in Figure 1. The dotted box in Figure 2 is the merged dense Merged_Dense(Batch_size,Param) of batch size = 32 with the following hyper-parameters:

(10) $$Merged\_Dense\left(32,564\right)=Conc(D_{Speech}\left(32,500\right),D_{Text}\left(32,64\right))$$

**Figure 3:**
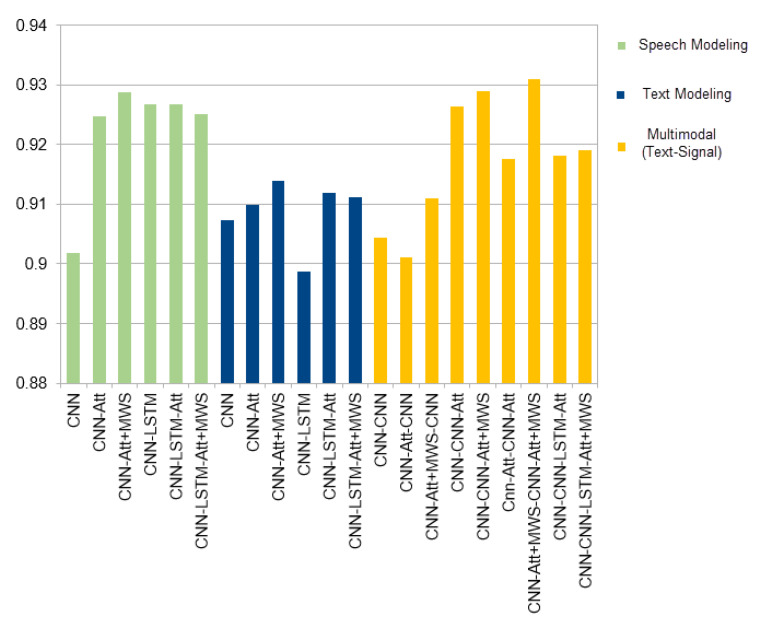
Modeling Approaches.

The results reported in Table 4 and Figure 3.

As shown in Figure 3, the multimodal approach provides a better classification accuracy by combining the CNNs-attention model for speech features and the CNNs-attention model for text features; both are implemented with the MWS function instead of the Softmax function. The Multimodal MWS approach had an improvement of 0.22% for modeling speech and 1.7% for modeling text. The accuracy of the multimodal classification for MWS was slightly better than that of Softmax by 1.34% for the same model. Findings reveal that the multimodal approach improves previous approaches and not combined models. However, we propose several lines in which this study could be extended for higher classification accuracy: (1) extending the vocabulary of the text model, in case of using the automatic transcription system, to reduce the Out Of Vocabulary (OOV) impact on productivity measurement, (2) improving the text model using pre-training approaches, i.e., Glove and BERT [38,39]. However, the previous pre-trained models do not support the Arabic language in the call centers domain, which requires more effort for data collection and training, (3) investigating other multimodal approaches like Coordinated-representation of structured space for merging the models [34].

Table 5 summarizes the results using MWS and Softmax functions for the models with the highest accuracy.

## 5. Conclusions

The automatization of the call center’s performance measurement is a critical task due to subjective evaluation. A novel method is proposed based on the multimodal approach by merging the speech and text models. The experiment was conducted over seven hours of speech at the real estate call center. In the study, 65 features of speech were applied using the Open-smile feature extraction instead of MFCC, and a significant improvement of 8.4% was achieved. The deep learning approaches for learning text improved the accuracy by 8.7% compared with the generative and discriminative approaches. The final multimodal approach achieved is 93.1%, which was an approximate improvement of about 1.7% over text classification and about a 0.22% improvement over speech processing. The Max weights similarity (MWS) method gave a minor improvement compared with the Softmax function, which is recommended for further investigation over different domains. It is recommended that future researchers extend this study by using the Bert context extraction for modeling text. Besides, applying various multimodal approaches in the early stages is worth investigating to improve the classifications.

## Figures and Tables

**Table 1:** 65 provided Low-Level Descriptors (LLD).

54 spectral LLD
RASTA-style auditory spectrum
MFCC 1–14
Spectral energy
Spectral Roll Off Point
Entropy, Spectral Flux, Skewness, Variance, Kurtosis,
Slope, Harmonicity, Psychoacoustic Sharpness
7 voicing related LLD
Probability of voicing, F0 by SHS - Viterbi smoothing
Jitter, logarithmic HNR, Shimmer
PCM fftMag spectral Centroid SMA numeric
4 energy related LLD
Sum of auditory spectrum
Sum of RASTA-style filtered auditory spectrum
RMS Energy
Zero-Crossing Rate

**Table 2:** Accuracy (Speech Processing) comparison.

Speech Accuracy % per Model Type		
**Classification Method**	**Type**	**Accuracy**
CNNs	MFCC	82.7%
CNNs-Attention	MFCC	84.27%
CNNs-BiLSTMs	MFCC	83.55%
CNNs-BiLSTMs-Attention	MFCC	83.54%
CNNs	LLD	90.1%
CNNs-Attention	LLD	92.48%
CNNs-Attention + MWS	LLD	92.88%
CNNs-BiLSTMs	LLD	92.67%
CNNs-BiLSTMs-Attention	LLD	92.68%
CNNs-BiLSTMs-Attention + MWS	LLD	92.25%

**Table 3:** Accuracy (Text Processing) comparison.

Accuracy % per Model Type		
**Classification Method**	**Type**	**Accuracy**
Naive Bayes	Bag of words	67.3%
Logistic Regression	Bag of words	80.76%
Linear Support Vector Machine (LSVM)	Bag of words	82.69%
CNNs	Word Embedding	90.73%
CNNs-Attention	Word Embedding	90.98%
CNNs-Attention+MWS	Word Embedding	91.4%
CNNs-BiLSTMs	Word Embedding	89.87%
CNNs-BiLSTMs-Attention	Word Embedding	91.19%
CNNs-BiLSTMs-Attention+MWS	Word Embedding	91.12%

**Table 4:** Accuracy (Multimodal models) comparison.

Multimodal Accuracy % per Model Type		
**Text Model**	**Speech Model**	**Accuracy**
CNNs	CNNs	90.44%
CNNs-Attention	CNN	90.1%
CNN	CNNs-Attention	92.63%
CNN	CNNs-Attention + MWS	92.9%
CNN-Attention	CNNs-Attention	91.76%
CNN-Attention + MWS	CNNs-Attention + MWS	93.1%
CNNs	CNNs-BiLSTMs-Attention	91.8%
CNNs	CNNs-BiLSTMs-Attention + MWS	91.9%
CNNs-Attention	CNNs-BiLSTMs	90.36%
CNNs-Attention + MWS	CNNs-BiLSTMs	91.1%
CNNs-Attention	CNNs-BiLSTMs-Attention	91%
CNNs-Attention + MWS	CNNs-BiLSTMs-Attention + MWS	91.1%

**Table 5:** The Table Compares the MWS method with Softmax used in Attention Layer.

MWS vs. Softmax—Accuracy Improvement%			
**Method**	**Speech Model**	**Text Model**	**Multimodal**
Softmax	92.68%	90.98%	91.76%
MWS	92.88%	91.4%	93.1%
Delta	0.2%	0.42%	1.34%
